# A “Novel” Protocol for the Analysis of Hydroxycinnamic Acids in Leaf Tissue of Chicory (*Cichorium intybus* L., Asteraceae)

**DOI:** 10.1100/2012/142983

**Published:** 2012-12-05

**Authors:** Meriem Bahri, Philippe Hance, Sébastien Grec, Marie-Christine Quillet, Francis Trotin, Jean-Louis Hilbert, Theo Hendriks

**Affiliations:** Université Lille 1, Sciences et Technologies, UMR INRA 1281 “Stress Abiotiques et Différenciation des Végétaux Cultivés”, GIS GENOCHIC, Bâtiment SN2, 3ème Étage, 59655 Villeneuve d'Ascq Cedex, France

## Abstract

A “novel” protocol is presented for easy and reliable estimation of soluble hydroxycinnamate levels in *Cichorium intybus* L. leaf tissue in large-scale experiments. Samples were standardized by punching 6 discs per leaf, and hydroxycinnamates were extracted by submerging the discs in 80% ethanol with 5% acetic acid for at least 48 h in the darkness at 4°C. Residual dry mass of the discs was used for *a posteriori* correction of compound levels. Chlorophyll was eliminated by chloroform, and the aqueous phases were transferred to microplates, dried, and dissolved in 50% methanol for HPLC analysis and storage. An HPLC program of 8 min was developed for the analysis of the extracts. Comparisons with extractions of liquid nitrogen powders indicated that the novel extraction method was reliable. No degradation of the major hydroxycinnamates—caftaric, chlorogenic, and chicoric acids—was observed, during maceration at ambient temperatures, or after storage for 1 year.

## 1. Introduction

Plants produce a wide array of secondary metabolites that play important roles in their interactions with their biotic and abiotic environments [1]. Hydroxycinnamates or phenylpropanoids (C6-C3 compounds), that is, ferulic, coumaric, caffeic, and sinapic acids, and their derivatives, are among the most widely distributed plant secondary metabolites and are precursors for the synthesis of many other molecules such as flavonoids, tannins, and lignin. They are particularly abundant in cereals, legumes, oilseeds, fruits, vegetables, and beverages [2, 3], and their occurrence, as well as their antioxidant activities, has been studied in relation to their proposed health benefits [3, 4].

The biosynthesis of hydroxycinnamates from phenylalanine via the phenylpropanoid pathway, and its genetic control, has been well studied in species like *Arabidopsis*, tomato, and sweet potato [5–7]. In contrast, despite their importance in terms of the number of species present in the wild and used by man [8], knowledge on the genetic control of this metabolic pathway in Asteraceae species is still limited, especially concerning the biosynthesis of hydroxycinnamates specific for this plant family, like caftaric and chicoric acid (e.g., [9–11]).

Chicory (*Cichorium intybus *L.) is an Asteraceae (Compositae) species belonging to the Cichoriinae, a subclade of the Cichorieae (Lactuceae) [12]. Appreciated as a medicinal plant for centuries in several civilizations [13], chicory is nowadays cultivated for numerous food and feed applications (see Cadalen et al. [14] for references). Hydroxycinnamic acids and flavonoids have been studied in chicory, particularly in leaf chicories [15–21]. To understand the genetic control of the metabolism of these molecules in chicory, one strategic approach is to identify quantitative trait loci (QTL) for the level of hydroxycinnamates in leaf tissue. A genetic map for chicory containing over 400 molecular markers is available [14], and initial results have indicated the existence of quantitative variation for the level of hydroxycinnamates and antioxidant activity in one of the progenies used for map construction. Though this concerns a progeny of root chicory, the results may be of interest in breeding programmes for all *C. intybus* cultigroups. Reliable methods of extraction and analysis of polyphenolic compounds in chicory have been reported with most of them being labour intensive and time consuming and require relative large quantities of material [16, 17, 19]. These methods are not well adapted when rapid sampling of large numbers of plants in the field or in a greenhouse is required, for instance to prevent developmental (and/or environmental) differences between the first and last plants sampled. In this paper, we describe a “novel” protocol that allows easy, practical, and reliable estimation of soluble hydroxycinnamate levels in chicory leaf tissue that can be scaled up in the light of QTL analyses requirements.

## 2. Materials and Methods

### 2.1. Plant Material

The studied plants were K59 and K28, two industrial chicory genotypes selected from the improved Hungarian landrace population “Koospol” (Florimond-Desprez, Cappelle-en-Pévèle, France) and plants of the F1 progeny K59 × K28 [14]. The original plants had been cloned by cutting, and the clones were grown in an unheated glasshouse under natural light conditions. All the studied plants were in vegetative state. 

### 2.2. Chemicals

All solvents used were of HPLC grade quality from VWR (West Chester, PA, USA). Authentic standards of caffeic, caftaric (*O*-caffeoyltartaric acid), and chlorogenic acid (5-*O*-caffeoylquinic acid) were purchased from Sigma-Aldrich (St. Louis, MO, USA). Chicoric acid (di-*O*-caffeoyltartaric acid) was supplied by ChromaDex, Inc. (Santa Ana, CA, USA). 

### 2.3. Extraction of Phenolic Acids

Two leaves (per plant) of comparable physiological age, that is, at similar, but opposite, positions on the plant, were sampled. From each leaf, three pairs of discs (diameter 0.9 cm) from the upper, middle, and lower parts, respectively, were collected in a 1.7 mL reaction tube (M*μ*lTI, Sorenson Bioscience Inc, Salt Lake City, UT, USA), by using its lid as a card puncher. The discs of each pair were taken on the both sides of the midvein. 

#### 2.3.1. “Novel” Method

Immediately after sampling, the discs were submerged in 500 *μ*L of 80% ethanol and 5% acetic acid and allowed to macerate for at least 48 h at 4°C in darkness. Thereafter, the discs were removed and dried to determine the residual dry weight, used for *a posteriori* correction of quantitative data. 

#### 2.3.2. “Conventional” Method

After sampling, tubes containing the discs were submerged in liquid nitrogen and the discs were crushed in the tube with a close-fitting pestle. The powder obtained was macerated for 1 hour at 4°C in darkness with 500 *μ*L of 80% ethanol and 5% acetic acid. After centrifugation (2 min, 11180 g, 4°C), the supernatant was transferred to a new tube.

For both methods, 100 *μ*L water and 500 *μ*L chloroform were added to each extract, and after vortexing and centrifugation (2 min, 11180 g, 4°C), 150 *μ*L of the chlorophyll-free aqueous phase was transferred to a well of a 96-well microplate (0.2 mL, Thermo-Fast 96, Low Profile, Thermo Scientific, UK), dried under vacuum, and dissolved in 100 *μ*L 50% methanol. The microplates were sealed (Thermo scientific, UK) and stored at −20°C for future analyses.

### 2.4. HPLC Analysis of Hydroxycinnamates

HPLC analyses were performed on a Prominence Shimadzu System (Palaiseau, France) equipped with a photodiode array UV-vis detector SPD-M20A (Shimadzu System). Standards were used to identify peaks by retention times and UV-vis spectra.

Initial analyses were carried out using a program of 36 minutes on a 150 × 4, 6 mm, 5 *μ*m LiChrospher RP-18 column (Phenomenex, USA), with a 4 mm × 3 mm guard column. 6 *μ*L of extract is injected and eluted with water (solvent A) and acetonitrile/2% acetic acid (solvent B). This method was adapted from previously described protocols [16, 22]. The column temperature was 25°C, and the flow rate was maintained at 1 mL·min^−1^. A multistep linear gradient was applied to elude the sample (Table 3). The column was equilibrated for 5 minutes between injections.

A shorter multistep linear gradient was optimized to reduce the time of analysis (Table 3). The 8-minute program was achieved on a 75 × 4 mm, 4 *μ*m LiChrospher RP-18 column (Phenomenex, USA), with the guard column described previously. A 6 *μ*L sample of each extract was analyzed. Solvents were A: water/1% acetic acid and B: acetonitrile/2% acetic acid. Flow rate was 1.8 mL·min^−1^, oven temperature, 55°C. Gradient steps are summarized in Table 3. The column was equilibrated for 2.8 min between each injection.

#### 2.4.1. LC/MS Analysis

LC/MS analyses were conducted by a Quattro II tandem quadrupole mass spectrometer (Waters Micromass, Manchester, UK) fitted with an electrospray ionization (ESI) source after separation by LC (Hewlett-Packard Model HP 1100, Agilent, Palo Alto, CA, USA). Data were acquired with MassLynx 4.0 data software. Hydroxycinnamate standards were dissolved to a concentration of 0.1 mg·mL^−1^. The mass spectrometer was operated in negative mode, except for chlorogenic acid. The source was maintained at 120°C. ESI interface was operated with a capillary voltage of 3.5 kV. The cone voltage was set at 30 V. Nitrogen was used as nebulization and drying gas. ESI mass spectra were acquired by scanning MS over the *m*/*z* range 240–700.

#### 2.4.2. MS/MS Analysis

MS/MS analyses were performed by transmitting the appropriate precursor ion through MS (*m*/*z* 311) to the collision cell. The collision gas used was argon with an *m*/*z* 311 collision energy of 15 eV.

### 2.5. Test of Variability Induced by the Manipulators

Four inexperienced testers without any relation to the experiment were instructed to pierce six discs from each of, in total, 24 chicory leaves at three defined places (bottom, middle, and top of the leaves). The discs were macerated with 80% ethanol and 5% acetic acid for one week, removed, and dried. Subsequent protocol steps were followed as described. The four results for each of the 24 leaves were statistically compared by ANOVA, according to the following model:

(1) $$\begin{array}{c} Y=\text{mean}+\text{tester}+\text{leaf}+\text{error}, \end{array}$$

where “mean” is the constant, “tester” measures the effects of having 4 different manipulators, “leaf” accounts for differences between 24 samples, and “error” is the residual term. 

### 2.6. Statistical Analyses

Statistics were conducted with Systat 12 Version 12.00.08, SPSS Inc., Chicago, IL, USA. Differences between samples were tested using ANOVA. 

## 3. Results and Discussion

### 3.1. Comparison between “Novel” and a “Conventional” Extraction Method and Identification of the Major Hydroxycinnamic Acids

Freezing and crushing fresh leaves in liquid nitrogen is a classical approach in polyphenolic extraction. This is generally followed by solvent extraction comprising selected variations with regards to solvent systems and time and manner of maceration. A critical precaution in the “conventional” method is to prevent the tissue or the powder to absorb humidity, which is liable to reactivate liberated enzymes that may modify or denature compounds of interest, like polyphenol oxidases [23]. The process of thawing is therefore common prior to extract being submerged in the maceration solution. In contrast, the extraction step in the “novel” protocol is based on combining a rapid fixation of tissue structure and proteins, as in preparations of tissues for histological analyses, and the liberation by diffusion of compounds, soluble in the maceration solution, in our case hydroxycinnamates.

**Figure 1:**
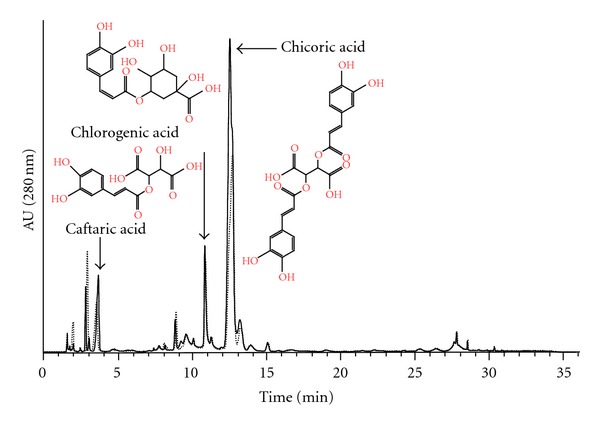
Chromatographic profiles of chicory leaves at 280 nm. Dotted line and continuous line represent HPLC profiles obtained for the same fresh leaf tissue area with a “conventional” liquid nitrogen grinding and the “novel” method, respectively.

To compare the efficacy of the “novel” method with the “conventional” extraction method, samples obtained by extracting equal amounts of leaf tissue by the two different methods were analysed. As the aim is to study hundreds of plants for genetic analysis, the fresh material was reduced to 6 discs (~70 mg FW) punched from upper, middle, and lower parts of a leaf with the lid of a 1.5 mL reaction tube. The maceration solution was 80% ethanol and 5% acetic acid, chosen as the most efficient solvent in the panoply commonly proposed to extract polyphenols. Similarly, chloroform turned out to be a judicious solvent to eliminate chlorophylls and to preserve molecules of interest (data not shown). HPLC chromatograms recorded at 280 nm showed similar profiles for the two methods, suggesting that the “novel” method is as efficient as the conventional one (Figure 1).

In the chromatograms, three main peaks were attributed to caftaric acid, chlorogenic acid, and chicoric acid by comparing their retention time and UV spectra with that of standard compounds, and their identity was confirmed by LC/MS analysis. The caftaric acid molecular ion was detected at *m*/*z* 311 (MW 312), and MS/MS analysis showed the presence of two main fragment ions, at *m*/*z* 179 and 149, corresponding to caffeic acid and tartaric acid, respectively, as well as a smaller signal at *m*/*z* 135, corresponding to the decarboxylation product of caffeic acid. The absence of the ion *m*/*z* 623 in MS/MS chromatograms, that would correspond to a dimer of caftaric acid, suggests that it is *cis*-caftaric acid [24]. The molecular ion [M+H]^+^ of chlorogenic acid was detected at *m*/*z* 355 (MW 354), and the ion [M-H]^−^ of chicoric acid at *m*/*z* 473 (MW 474). The data reveal that chicoric acid was the most abundant compound, followed by caftaric and chlorogenic acids (Figure 1), thereby agreeing with results from previous studies on other varieties of *C. intybus* L. [17, 19, 22] or *C. endivia* L. [16]. 

Quantitative differences between the “novel” method and the “conventional” extraction method were investigated by comparing the levels of the most abundant compounds obtained from 6 leaves taken from 3 different plants (Table 1). Pair-wise comparisons of samples, that is, between two similar leaves from the same plant (samples 1 and 2, 3 and 4, and 5 and 6), showed reproducible differences in the levels of caftaric, chlorogenic, and chicoric acids extracted by the two methods. This indicated that the “novel” extraction method is at least as reliable as the more conventional method.

In general, the overall levels of hydroxycinnamates extracted with the “novel” method were higher than those in the conventional method (Table 1). One possible cause for these differences may be the time of maceration in the “conventional” method in comparison to the “novel” method, that is, 1 h versus 48 h, respectively. The selective influence on chicoric acid, characterized by major quantitative changes in comparison to caftaric and chlorogenic acids (Table 1), is unclear. In this respect, it is interesting to note that in an assay referred to as a validated method to determine caftaric acid, chicoric acid, chlorogenic acid, and echinacoside content in plant materials of *Echinacea* species, samples are prepared by extracting 0.125 g powdered material by shaking (ultrasonic) in 25 mL 70% (v/v) ethanol for 15 min only (Method 106.000 of the Institute for Nutraceutical Advancement [25, 26]). An alternative explanation may be that levels of chicoric acid in the conventional method extracts are reduced by the activity of enzymes released from the tissue upon grinding and maceration. As shown for *Echinacea purpurea* (L.) Moench preparations [23], chicoric acid does not seem to be stable under conditions where oxidative degradation of caffeic acid derivatives are inhibited, and it was found that under these conditions an esterase hydrolyzing the ester bonds between tartaric acid and caffeic acid is still active. Our results (Table 1) did not allow discriminating between these two possibilities. Further research is required to resolve this difference between the two extraction methods. Nonetheless, extraction by the “novel” method seems to prevent degradation of the three selected compounds, a mechanism most probably linked to the precipitation of enzymes within the tissue and therefore making them unavailable in the maceration solution. 

Chromatograms of some samples extracted by the “novel” method show in some instances a very small peak corresponding to the position of caffeic acid. However, in the material analysed so far, caffeic acid levels remained below the level of detection and quantification. Caffeic acid was identified in leaves of root chicory “Magdeburg” [27], and it was also detected in some salad chicories, like Chioggia, Grumolo, Castelfranco, and Verona [18]. However, in other cultigroups, caffeic acid was not detected in free form but only esterified with quinic or tartaric acid [16, 17, 19, 22, 24, 27]. Characterization of the other minor peaks present in the chromatograms is in progress. In comparison with previous investigations in chicory varieties, the presence of gallic acid, protocatechuic acid, and other caffeoyl quinic and tartaric derivatives can be expected. Flavonoids identified in green chicory varieties are kaempferol, quercetin, luteolin, and apigenin, often bound to glucose, or a glucuronide [16–20, 22, 24, 27]. Nonetheless, a focus on caftaric, chlorogenic, and chicoric acids for QTL analysis is justified, since they are the main phenolic compounds in chicory leaves irrespective of the age of the plant or culture conditions, representing ~60% of total phenolic acids detected in our extracts.

### 3.2. Test of Variability Induced by the Manipulators

**Figure 2:**
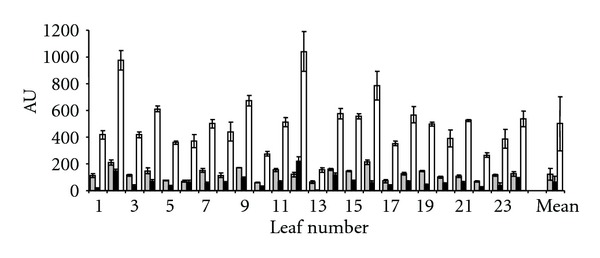
Histogram representing the contents (arbitrary unity) in caftaric acid (grey), chlorogenic acid (black), and chicoric acid (white) for each of the 24 different leaves analyzed by four inexperienced testers. The differences obtained between the manipulators are showed by the standard deviations (no significant differences; see Table 2).

A possible source of variation in the “novel” protocol could be the sampling of the 6 discs per leaf by the experimenter. To test this, four inexperienced persons were instructed to sample 6 discs of 24 leaves, 2 at the top, 2 in the middle, and 2 at the base, and each pair was separated by the midvein. Thereafter the discs were extracted as described, the extracts analysed by HPLC, and the results obtained for each of the 24 leaves sampled by the 4 different experimenters were compared (Figure 2). ANOVA tests indicated that there were no significant differences between the results obtained by the 4 testers (*P* > 0.05, italic values, Table 2). As each leaf was sampled 4 times (i.e., 24 discs per leaf), and thus the positions at the top, middle, or lower portion of the leaf at which the discs were taken differed for each of the experimenters, this indicated that the method allows reliable sampling by inexperienced interchangeable persons.

### 3.3. Optimization and Characteristics of the “Novel” Protocol

To further speed up sampling in the “novel” protocol, discs were directly submerged in maceration solution without determination of fresh mass. Instead, the dry mass of the discs was determined after extraction. As investigated in a separate experiment on 6 different plants, dry mass prior extraction represented on average 15.7% ± 1.5 of disc's fresh mass, whereas this was 12.8% ± 0,9 for the residual dry mass after extraction of the discs. The constancy between fresh mass and the dry mass after extraction thus allows a correction of the hydroxycinnamate levels for the amount of tissue extracted.

To evaluate the influence of temperature on extraction, two temperatures of maceration were tested on 12 samples: 4°C and 22°C. Pair-wise comparison of HPLC profiles showed that they overlapped, and ANOVA tests on peak areas revealed no significant differences (*P* > 0.05) between the two temperatures. This seems to confirm a lack of enzymatic degradation of hydroxycinnamate compounds in extracts obtained by the “novel” protocol.

With regards to the maceration time, 4 samples from the same leaf were macerated for 24, 48, 72, or 96 hours. No qualitative differences were detected between the samples. At the quantitative level, 24 hours of maceration were slightly less efficient than the more prolonged periods of maceration. Taken together, these observations indicate that for the “novel” method, no strict temperature conditions are necessary and that maceration time can be adapted according to the number of plants to be analysed and to the number of persons conducting the work.

Though maceration takes a relative long time, this is compensated for by its ease, and by the high number of samples that can be treated at the same time. Furthermore, the number of disposables used is low, and there is no need for additional transfers and treatments that may require specialized (expensive) equipment when a large numbers of sample are to be processed simultaneously. Considering the number of samples needed for quantitative genetic analyses, the storage in micro-well plates is convenient, and the microwell plate format can also be used for further analyses of the samples, that is, the evaluation of antioxidant capacity using multiantioxidant assay systems (e.g., DPPH•, ABTS•^+^, or FRAP assays [28–30]).

To investigate the stability of samples conserved in 50% methanol, 36 extracts were reanalysed one year after storage at −20°C. Comparisons of HPLC profiles showed that quantitative differences between the two analyses were within the range of standard errors. This confirmed once more the absence of enzymatic degradation and indicated that samples can be safely stored in 50% methanol for a long time at −20°C before analysis.

### 3.4. Improvement of HPLC Program

**Figure 3:**
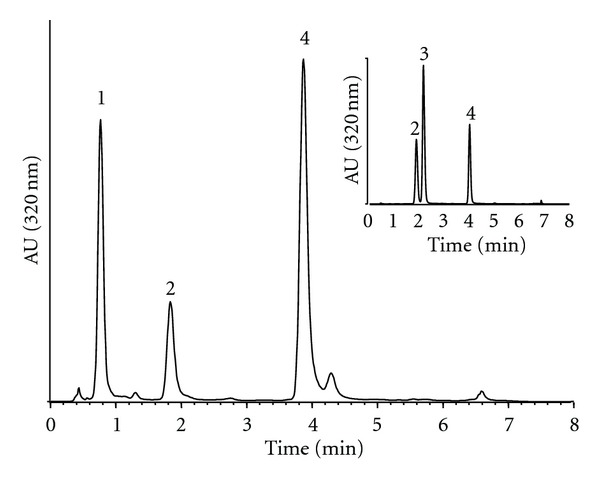
HPLC profile of chicory leaves with the “short program,” using the “novel” method. Peak identifications: (1) caftaric acid, (2), chlorogenic acid, (3) caffeic acid (insert), and (4) chicoric acid. Chromatogram was recorded at 320 nm.

Having established the conditions for the extraction of hydroxycinnamic acids in the “novel” protocol, the HPLC method was adapted to reduce the time of analysis. The results obtained with the initial method indicated that the separation on C18 reversed phase silica gave reliable results upon analysis of the chicory extracts. To achieve faster analysis, separation was carried out on a 75 mm long LiChrospher RP-18 column, rather than the 150 mm one used in the previous method, and elution solvent composition and proportion were adjusted (Table 3). This resulted in a reduction of the time of HPLC-DAD analysis to 8 min without loss of baseline separation of the components of interest (Figure 3).

Pure hydroxycinnamic acids and chicory extracts were used to validate the adapted HPLC analytical method. Sequential analyses of standards (chlorogenic, caffeic, and chicoric acids, cf. insert Figure 3), diluted series of chicory leaf extract, and chicory leaf extract spiked with standards, each followed by a sample of extraction solution, were repeated 3 times per day on 6 different days. After the comparison of the UV spectra, no endogenous interference at the retention time of the hydroxycinnamic acids was detected in the blanks following each analysis from sample extract. Overall, no interfering peaks were observed from the matrix extract, and peak purity was higher than 90% for any eluted compound. Resolution was higher than 1.5 for all peaks. Finally, robustness of the method was tested by successively decreasing the parameters temperature, flow rate, and acetic acid concentration by 10% and 20%. None of these adjustments resulted in significant differences (*P* > 0.05), and similarity for quantitative values was higher than 95%.

To quantify hydroxycinnamic acids levels in extracts, an external standard method based on peak areas of the eluted compounds was used. Working range concentration for each compound was estimated from dilution series of a sample extract, and chlorogenic acid was used to construct a five-point calibration curve (0.5–150 *μ*g·mL^−1^) applying a least-squares regression analysis. A good linear relationship was obtained with a correlation coefficient of 0.997. The LOD (limit of detection: concentration of compound yielding a signal/noise ratio of 3 : 1) and the LOQ (limit of quantification: concentration of compound yielding a signal/noise ratio of 10 : 1) obtained, 0.07 *μ*g·mL^−1^ and 0.24 *μ*g·mL^−1^, respectively, were suitable for quantitative analyses of hydroxycinnamic acid levels in chicory extracts. Repeatability and reproducibility for retention times and of quantifications were higher than 97%. Accuracy was assessed by analyzing chicory extract before and after spiking with three different concentrations of standards (0.06, 0.12, and 0.25 *μ*g·mL^−1^), and quantities recovered ranged between 96% and 108%.

In conclusion, the estimation of soluble hydroxycinnamic acid levels in chicory leaf tissue by the protocol presented in this study proved to be easy and reliable and allows a rapid screening of the phenolic acids content in leaves of different chicory cultigroups, and possibly also in other leafy vegetables or pharmaceutical herbs (e.g., [31, 32]). As the extraction part of the “novel” protocol does not require liquid nitrogen to preserve the material sampled, it is particularly suited for sampling large numbers of plants in a greenhouse or in the field. Considering the number of samples needed for quantitative genetic analyses, the storage in micro-well plates is convenient, and the micro-well plate format can also be used for further analyses of the samples, for example, the evaluation of antiradical capacity. Preliminary results indicated that the “novel” protocol is a useful tool in the identification of QTL for the level of hydroxycinnamates in chicory leaf tissue. After sampling near 1800 leaves, followed by quantitative analyses using the new HPLC protocol, average concentrations were determined as 246 ± 120 mg·kg^−1^ FW for caftaric acid, 130 ± 92 mg·kg^−1^ FW for chlorogenic acid, and 370 ± 202 mg·kg^−1^ FW for chicoric acid.

## Figures and Tables

**Table 1:** Levels of caftaric, chlorogenic and chicoric acids in extracts obtained by the “novel” method and a “conventional” method. Similar amounts of tissue sampled from 2 comparable leaves from 3 different chicory plants were extracted by the two methods, and quantities of the hydroxycinnamate compounds in the extracts were determined by HPLC (see text for details). Content are expressed in *μ*g/cm^2^ of fresh leaf tissue area.

Sample	Compound	“Novel"		“Conventional"		Novel/conventional (%)
		*μ*g/cm^2^	%	*μ*g/cm^2^	%	
1	Caf	7.1	26.2	7.6	33.4	92.6
	Chlo	5.8	21.7	5.8	25.2	101.4
	Chic	14.1	52.1	9.5	41.4	148.4
	Sum	27	100	22.9	100	117.9

2	Caf	6.8	25.1	7.6	33.2	89.9
	Chlo	6.2	22.9	5.7	24.8	109.7
	Chic	14.2	52.1	9.6	42	147.6
	Sum	27.2	100	22.8	100	119

3	Caf	13.8	19.1	16	28.7	86
	Chlo	15.8	21.9	16.6	29.9	94.6
	Chic	42.5	59	23	41.4	184.3
	Sum	72	100	55.7	100	129.2

4	Caf	13.9	18.9	16	28.6	87.2
	Chlo	15.7	21.2	16.7	29.9	93.8
	Chic	44.2	59.9	23.1	41.5	191.1
	Sum	73.8	100	55.8	100	132.2

5	Caf	11.3	17.9	7.8	18.7	145.5
	Chlo	16.8	26.5	13.9	33.2	121.2
	Chic	35.3	55.7	20.1	48.1	176.1
	Sum	63.5	100	41.7	100	152.1

6	Caf	11.4	17.9	8.8	20.3	128.8
	Chlo	16.8	26.4	14.2	32.5	118.8
	Chic	35.5	55.7	20.6	47.2	172.3
	Sum	63.7	100	43.6	100	146.1

**Table 2:** ANOVA of caftaric, chlorogenic, and chicoric acid contents estimations of 24 chicory leaves extracted by 4 different testers using the “novel” method.

Variables and source of variation	Degree of freedom	Mean square	*F*-ratio	*P* value
Caftaric acid				
Tester	3	0.005	2.67	*0.054 *
Leaf	23	0.096	49.63	<0.001
Error	68	0.002		
Chlorogenic acid				
Tester	3	0.004	0.266	*0.850 *
Leaf	23	0.376	23.11	<0.001
Error	68	0.016		
Chicoric acid				
Tester	3	0.005	0.869	*0.461 *
Leaf	23	0.118	21.75	<0.001
Error	68	0.005		

**Table 3:** Solvent gradients in the “long” and “short” HPLC analyses of hydroxycinnamates in extracts of chicory leaf tissue. Solvent A consisted of water for the “long” program and water/1% acetic acid for the “short” program.

Long program (36 minutes)		Short program (8 minutes)	
Time (min)	Solvent A (%)	Time (min)	Solvent A (%)
0	100	0	94
2	92	1.4	90
5	87	4.2	84.3
15	85	4.4	81
16	83.5	4.8	0
18	81.5	5.2	94
22	80		
26	0		
29	0		
31	100		
